# Low Serum Testosterone as a Poor Prognostic Marker in Urethral Stricture: A Single-Center Prospective Longitudinal Study

**DOI:** 10.7759/cureus.58895

**Published:** 2024-04-24

**Authors:** Soumya Mondal, Amitayu Jana, Debansu Sarkar

**Affiliations:** 1 Urology, Institute of Post Graduate Medical Education & Research, Kolkata, IND

**Keywords:** follicle stimulating hormone (fsh), luteinizing hormone (lh), non-stricture (ns), urethral stricture (us), lichen sclerosis (ls), thyroid-stimulating hormone (tsh)

## Abstract

Introduction

Androgens play a key role in modulating periurethral and preputial vascularity, cavernosal smooth muscle integrity, and penile growth. As a result, low testosterone may adversely affect the severity and outcome of urethral stricture patients. So, to find out the hormonal influence on the clinical outcome of urethral stricture we conducted a prospective longitudinal study at our institute.

Methods

The study was conducted at the Department of Urology, Institute of Post Graduate Medical Education & Research (IPGMER), Kolkata, India, from February 2023 to September 2023. This study was approved by the Institutional Ethics Committee at IPGMER, Kolkata with the approval number IPGMER/IEC/2023/436. Hormonal levels in patients with diagnosed non-traumatic urethral stricture were compared with patients without stricture. Patients with any overt hormonal abnormality or androgen-secreting tumor were excluded. A morning 10 cc blood sample was collected for testosterone, follicle-stimulating hormone, luteinizing hormone, and thyroid-stimulating hormone. The association of hormonal levels was measured in both groups and compared statistically. Any association of hypogonadism (testosterone <300 ng/dL) with respect to length, severity, and recurrence of urethral stricture was also studied.

Results

Forty patients with urethral stricture and same number of patients without stricture were included in the study. The mean testosterone level was found to be significantly low in patients with stricture (386 ng/dL vs 660 ng/dL). The age-wise distribution also showed low mean testosterone compared to patients without stricture. The incidence of hypogonadism is also found to be higher in stricture patients (47.5% vs 27.5%). It was also observed low testosterone is more prevalent in pan-anterior stricture (10/40) and long-segment stricture(>2 cm). Patients with stricture were also followed up for 6 months for recurrence of symptoms. Thirteen patients had recurrence. Patients with recurrence had significantly low serum testosterone (272 ng/dL vs 440 ng/dL).

Conclusion

Our study documented stricture patients with low serum testosterone have poor outcomes. Low testosterone level is strongly associated with longer stricture and increased risk of stricture recurrence.

## Introduction

Urethral stricture is one of the leading urological causes of male morbidity affecting the quality of life [1,2]. It results in a significant financial burden on our healthcare system [3,4]. The aetiologies documented are idiopathic, iatrogenic, traumatic, infective, and inflammatory (Balanitis Xerotica Obliterans). Idiopathic factor (31-44%) remains the major cause. Iatrogenic factors like instrumentation, traumatic catheterization, and hypospadias surgery also contribute to about 32% of urethral stricture [5-9]. However, the exact pathogenesis and molecular mechanism still need to be explored as the involvement of aforesaid factors does not always result in stricture. Hormonal interactions have a key role as androgens increase vascularity and healing by promoting angiogenesis [10]. Studies have exhibited an association of low testosterone with inflammation and fibrosis of hepatic, respiratory and cardiac tissues [11-13]. Androgens are also engaged in maintaining preputial vascularity, cavernosal smooth muscle integrity, penile growth, and improved wound healing through a receptor-mediated pathway [14]. Therefore, low testosterone may result in improper healing and exaggerated inflammation with fibrosis and stricture formation. The association of various hormonal interplay has hardly been studied in prospective or retrospective literature. We hypothesize an association between low testosterone and urethral stricture. Therefore, the purpose of the study is to find out any relation between serum testosterone level in urethral stricture and its severity and recurrence.

## Materials and methods

This comparative study was conducted in the Department of Urology of the Institute of Post Graduate Medical Education & Research (IPGMER), Kolkata, India, after obtaining ethical clearance approval from the IPGMER Oversight Committee (Institutional Ethics Committee) with the approval number IPGMER/IEC/2023/436.

Selection of cases and controls

Patients aged between 18 and 75 years and diagnosed with non-traumatic urethral strictures (idiopathic or Balanitis Xerotica Obliterans (BXO)) admitted in our urology department from February 2023 to September 2023 were included in the study. Patients were previously assessed by retrograde urethrography and urethroscopy. The non-stricture (NS) group comprised admitted patients in the department with symptoms other than any voiding difficulties, such as renal mass, pelvi-ureteric junction obstruction, bladder tumor, and renal and ureteric calculus. They were enquired of any voiding-related symptoms, history of sexually transmitted disease, and catheterization, and if present, were excluded from the study.

Exclusion criteria for both groups were patients with iatrogenic and traumatic urethral stricture like pelvic fracture urethral distraction defect, documented hormonal abnormalities such as carcinoma prostate on hormone replacement therapy or castrated, hypopituitarism, suspected primary testicular failure, testicular tumor, androgen-secreting tumors, pelvic irradiation for malignancy, history of steroid use and patients who did not want to be a part of the study.

Sample size

According to the study by Anger et al. [15], the expected prevalence of urethral stricture in the general population is 8.4%. Then, for an alpha risk of 5% and an estimated loss of 6.5%, with a power of 89% and a 95% confidence interval, the sample size was (4pq/L2)74 with 37 in each group. However, we extended our study to recruit 40 patients in each group.

Statistical analysis

For statistical analysis, data were entered into a Microsoft Excel spreadsheet and then analyzed by SPSS software (version 27.0; IBM Corp., Armonk, NY) and Graph Pad Prism version 5 (GraphPad Software, Boston, MA). Data had been summarized as the mean and standard deviation for numerical variables. Statistical analysis was carried out using the Chi-square test(χ) and the 't' test. Multivariate regression analysis was done to identify independent risk factors. The odds ratio (OR) was calculated for independent predictors of recurrence. P value ≤ 0.05 was considered statistically significant.

Measurement of exposure and outcome

After obtaining approval from the Institute of Post Graduate Medical Education & Research Oversight Committee (Institutional Ethics Committee) (approval no. IPGME & R/IEC/2023/436), the study tools were developed. Data collection was started after explaining the purpose of the study and obtaining informed consent from the patients. Data was maintained in an Excel sheet.

Participants were scheduled for morning 10 cc of blood sample collection in fasting conditions. Blood samples were sent to the institute’s hormonal assessment laboratory for assessment of testosterone (ng/dL), follicle-stimulating hormone (FSH; mIU/mL), luteinizing hormone (LH; mIU/mL), and thyroid-stimulating hormone (TSH; mIU/mL). All measurements were done in the same laboratory using the same methodology. Hormonal levels were calculated by spectrophotometric chemiluminescence immunoassay.

Patients with urethral stricture (US) were divided according to the site of involvement, such as penile, bulbar, bulbo-membranous, and pan-anterior stricture. After surgical intervention, patients were followed up in OPD for six months for voiding-related symptoms. Patients with symptoms were again evaluated by uroflowmetry, retrograde urethrography, and urethroscopy and diagnosed as recurrence.

The outcomes were compared statistically. Exposure variables were total testosterone, FSH, LH, and TSH. We also included age, BMI, and TSH as potential confounders. Normal ranges of testosterone, FSH, LH, and TSH were pre-set by the examining laboratory. We calculated mean testosterone, FSH, LH, and TSH values and compared them between US and NS groups. Multivariate analysis was carried out to find out any relationship between hormonal level with site and severity of US. Hypogonadism is defined as a testosterone level of less than 300 ng/dL. 

## Results

The mean age of patients in both groups was 45.05± 12.83 years (US) and 46.67± 13.54 years (NS) (p=0.423). BMI of both groups was similar (24.52 kg/m^2^ vs 23.64 kg/m^2^) (p=0.0054) (Table 1). Patients with US were found to have significantly low mean testosterone levels compared to the NS group(386 ng/dL vs 660 ng/dL) (p<0.0001) (Table 1). The incidence of low testosterone (T<300 ng/dl) was observed to be higher in the US (47.5%, n=40) than in the NS (27.5%, n=40) population. The comparison of mean testosterone between urethral stricture and non-stricture patients were found be statistically significant (p<0.0001).

**Table 1:** Mean distribution of age, BMI, testosterone, FSH, LH, and TSH level between urethral stricture and non-stricture group Data presented as Mean ± SD; p-value ≤ 0.05 was considered statistically significant. BMI: Body mass index; FSH: Follicle-stimulating hormone; LH: luteinizing hormone; TSH: Thyroid-stimulating hormone.

	Urethral stricture (n=40) (mean±SD)	Non-stricture (n=40) (mean±SD)	p-value
Age (years)	45.05± 12.83	46.67± 13.54	0.423
BMI (kg/m^2^)	24.52± 2.64	23.64± 2.44	0.005
Testosterone (ng/dL)	386.00 ± 225.60	660.82 ± 298.26	<0.0001
FSH (mIU/mL)	5.46± 2.98	5.47± 2.28	0.990
LH (mIU/mL)	4.40± 1.71	4.28± 1.88	0.776
TSH (mIU/mL)	2.16± 1.014	2.65± 1.285	0.064

The distribution of FSH (5.46±2.98 mIU/mL vs 5.47± 2.28mIU/ml, p=0.990), LH (4.40±1.71 mIU/ml vs 4.28± 1.88 mIU/ml, p=0.776) and TSH (2.16±1.01 mIU/ml vs 2.65±1.28 mIU/ml, p=0.064) values were almost similar between two groups.

**Figure 1:**
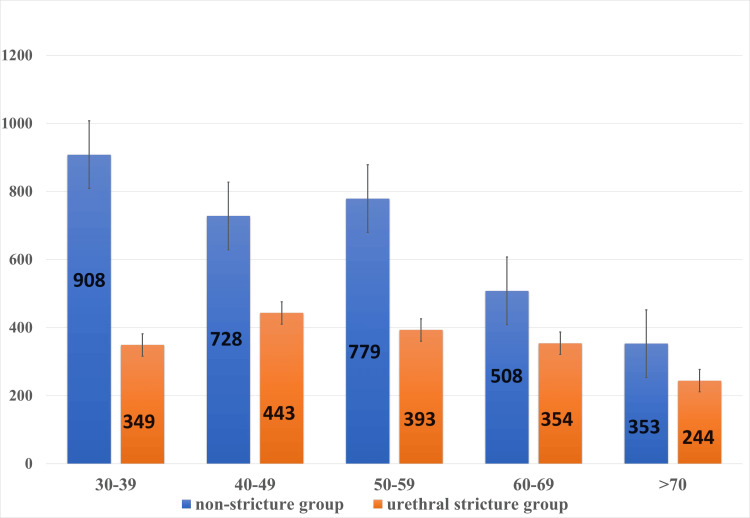
Age-wise distribution of mean testosterone level (ng/dl) between urethral stricture and non-stricture groups Data presented as mean

The association between urethral stricture and FSH, LH, and TSH was not statistically significant (p≥0.05). The age-wise distribution of mean testosterone level was significantly low in the US group compared to NS groups and found statistically significant (p=0.004) (p-value ≤ 0.05) (Figure 1).

Among the stricture patients (n=40), 10 were penile, 16 were bulbar, 3 were bulbo-membranous and 11 were pan anterior region strictures. Low testosterone level (<300 ng/dl) was observed in 19 stricture patients (47.5%, n=40). Hypotestosteronemia was more prevalent in pan-anterior (52.9%, n=19) followed by bulbar urethra (31.6%, n=19) (χ2=13.898, p=0.0076) (Table 2).

**Table 2:** Association between stricture site and testosterone Level Data represented as number of patients and percentage (%); p-value ≤ 0.05 was considered statistically significant.

Stricture	Testosterone		Total (n=40)	p-value	Chi-square [χ2]
	<300 ng/dl	>300 ng/dl			
Penile	2 (10.5)	8 (38.1)	10 (25)	0.0076	13.898
Bulbar	6 (31.6)	10 (47.6)	16 (40)		
Bulbo-membranous	1 (5.3)	2 (9.5)	3 (7.5)		
Pan anterior	10 (52.6)	1 (4.8)	11 (27.5)		
Total (n=40)	19 (47.5)	21 (52.5)	40 (100)		

In US patients, uroflowmetry mean Q max was 2.47± 3.44 ml/s in penile, 3.51± 3.89 ml/s in bulbar, 6.5±1.99 ml/s in bulbo-membranous and 7.04± 3.02 ml/s in pan anterior stricture, respectively (p=0.0201) (Table 3). Long segment stricture (>2 cm) was noted in 12 patients of which 10 patients had low serum testosterone (Table 4) and found statistically significant (p=0.0112) (χ=8.9775).

**Table 3:** Uroflowmetry values (Qmax) in the US group Data presented as Mean ± SD; p-value ≤ 0.05 was considered statistically significant; US: Urethral stricture.

Stricture site	Number of patients	Q-max (ml/sec)	p-value
Penile	10	2.47± 3.44	0.0201
Bulbar	16	3.51± 3.89	
Bulbo-membranous	3	6.5±1.99	
Pan anterior	11	7.04± 3.02	
Total (n=40)	40		

**Table 4:** Association between stricture length and testosterone Level Data represented as the number of patients (%); *Chi-square test; p-value ≤ 0.05 was considered statistically significant.

stricture length (cm)	testosterone		total(n=40)	p value	ch-square value [χ2]
	<300 ng/dl	>300 ng/dl			
<1	2 (10.5)	3 (14.3)	5 (12.5)	0.0112^*^	8.9775
1-2	7 (36.8)	16 (76.2)	23 (57.5)		
>2	10 (52.6)	2 (9.5)	12 (30)		
TOTAL	19	21	40 (100)		

**Figure 2:**
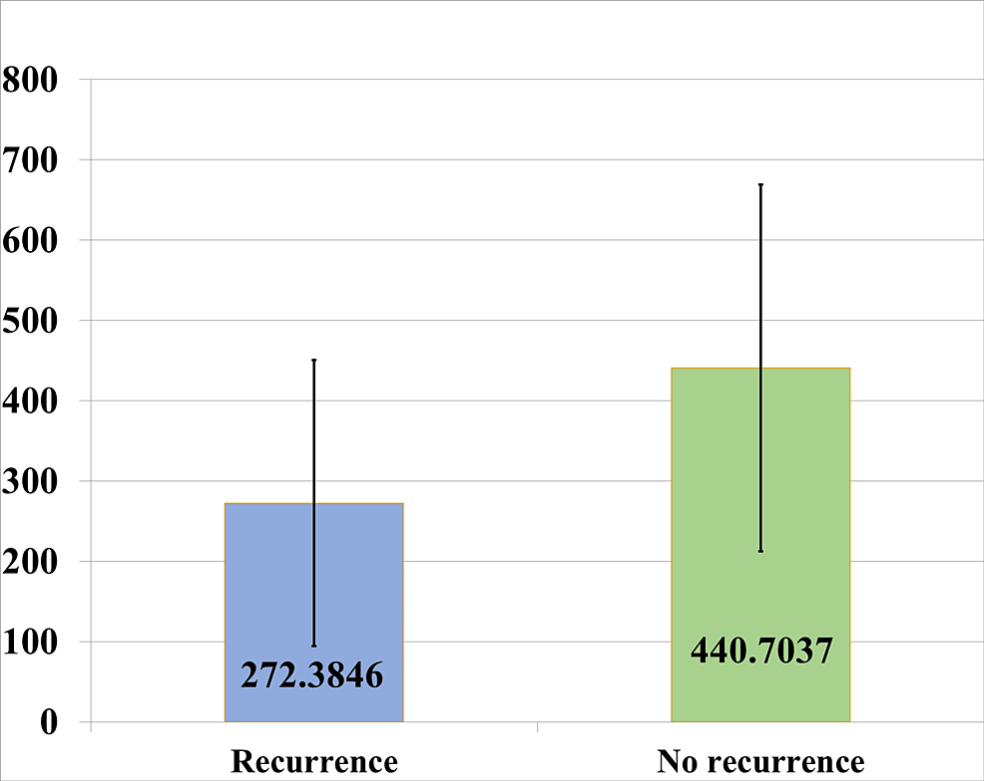
Distribution of mean testosterone level (ng/dl) with recurrence

After six months of follow-up, 13 (32.5%) patients had a recurrence and needed reintervention, and 27 patients were symptom-free. Patients with recurrence had significantly low mean testosterone (272.38 ng/dl vs 440.70 ng/dl) (p=0.0251) (Figure 2) (Table 5).

**Table 5:** Association of mean testosterone and recurrence Data presented as Mean ± SD; * Unpaired t-test; p-value ≤ 0.05 was considered statistically significant.

	No. of patients	Testosterone level (mean ±SD)	p-value
No recurrence	27	440.70± 228.36.	0.0251^*^
Recurrence	13	272.38± 178.05	
Total (n=40)	40		

Among 13 patients who had recurrence low serum testosterone (< 300 ng/dl) was noted in nine patients. The majority of them had pan anterior (5/13) and bulbar urethral stricture (2/13) (p=0.3266) (Figure 2).

**Figure 3:**
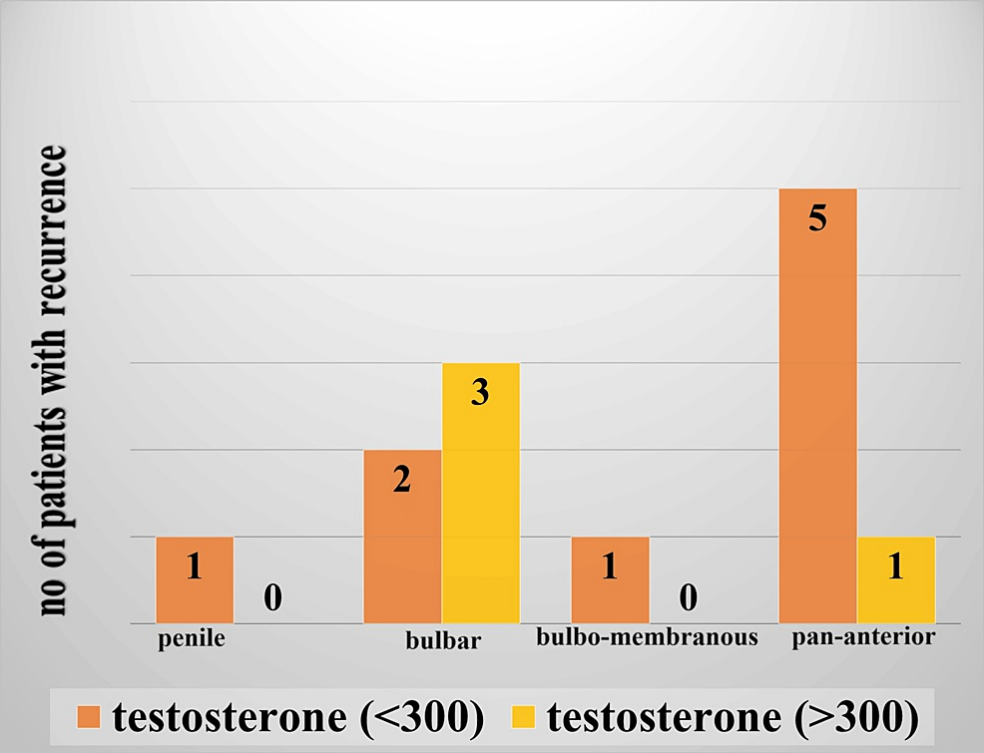
Relationship between site of recurrence and serum testosterone level

Binary logistic regression was performed to see the independent predictors of recurrence. Initially, all variables were assessed in a univariate model. Following that, all variables were put in a multi-variable model using the enter method, and then non-contributory variables were removed using the bidirectional step by the selection method. Finally, it was observed that testosterone level (OR=1.98, 95%CI=0.95-3.25, p=0.042) and length were a significant predictor of recurrence (OR=2.02, 95% CI=1.05-3.86, p=0.034) (Table 6).

**Table 6:** Multivariate analysis for independent variables Data presented as mean (standard deviation); *Odds ratio (95% CI); p ≤ considered statistically significant.

	No recurrence	Recurrence	Odds ratio (95% CI) *	p-value
BMI	25.9 (3.4)	25.2 (4.3)	0.76 (0.56-1.02)	0.063
Age	53.5 (12.8)	58.8 (12.6)	1.04 (0.98-1.10)	0.234
Testosterone (ng/dl)	440.7 (228.3)	272.38 (178)	1.98 (0.95-3.25)	0.042
Length of stricture (cm)	1.6 (1.1)	2.6 (1.8)	2.02 (1.05-3.86)	0.034

## Discussion

The exact etio-pathogenesis of urethral stricture has been poorly understood till now. Multiple etiologies have been described. Although factors like lichen sclerosis, urethritis, and iatrogenic injuries have a well-established pathogenesis, recurrences and clinical severity of stricture largely vary among patients. This may be due to an independent risk factor which is responsible for different clinical presentations and outcomes of urethral stricture. In our study mean age (45 years vs 46 years) and BMI (24.52 kg/m^2^ vs23.64 kg/m^2^) (p= 0.005)between US and NS groups were similar. Kirk et al. have exhibited an association between low testosterone, lichen sclerosis, and BMI [16]. TSH can alter testosterone levels by increasing serum hormone-binding globulin. TSH levels in both groups were similar (2.16 mIU/ml vs 2.65 mIU/ml). Age, BMI, and TSH can influence blood testosterone levels, thus being taken as confounding factors.

In our study, the mean testosterone level in US patients was lower than in NS patients (386 ng/dL vs 660 ng/dL, p-value=0.0001). Within stricture patients, 47.5% (n=40) had low serum testosterone (<300 ng/dl) compared to 27% (n=40) in the NS group. As serum testosterone levels vary with age, the mean testosterone levels of different age groups in both populations were compared and revealed to be significantly low in the stricture population compared to NS cases. Mean FSH (5.46 mIU/ml vs 5.47 mIU/ml), LH (4.40 mIU/ml vs 4.28 mIU/ml) were similar between two groups. Although we expected high FSH and LH as a result of negative feedback from low testosterone, values were within the normal range. This explains the possibility of underlying functional hypogonadism [17]. Among stricture patients who had low serum testosterone, the highest incidence was seen in pan anterior cases (52.5%, n=10/19) (p=0.0076). It was also supported by a study done by Spencer et al. [18]. Bivariate analysis showed that the incidence of low testosterone is more prevalent in long-segment stricture (>2 cm) (p=0.0112).

After the intervention, stricture cases were followed up for six months for recurrence. About 13 patients (32.5%, n=40) had stricture recurrence and needed re-intervention. Mean testosterone in patients who had recurrence was low compared to those who did not have recurrence (272.38 ng/dl vs 440.70 ng/dl) (p=0.0251). All the associations were statistically significant. It was observed low testosterone is more predominant in pan anterior stricture with more incidences of recurrence (Figure 3). Multivariate binary regression analysis showed serum testosterone and stricture length to be independent predictors of recurrence (Table 6). For every 100 ng/dl decrease of serum testosterone, the risk of recurrence increases by 1.98 times (1.98 95%CI=0.95-3.25, p=0.042). 

A study by Spencer et al. assessed preoperative serum testosterone among 115 stricture patients [18]. It was observed that 65 (56.5%, n=115) patients had low serum testosterone. They compared the data with the general population aged more than 18 years. Bonilla et al. recently documented a cross-sectional study of 120 stricture patients and 41 controls [19]. Mean testosterone was documented as significantly low in stricture patients (391 ng/dL vs 495 ng/dL) [19]. Testosterone level below the normal limit (<300 mg/dl) was observed more in urethral stricture (35.8%, n=123) than in control (14.6%,n=41)[ p<0.007]. Recently, Puche et al. conducted an assessment of testosterone (total- T, Free-T) among 149 patients with urethral stricture and 66 patients without any voiding-related symptoms [20]. The study showed mean total testosterone was significantly lower (394 ng/dL vs 488 ng/dL, p <0.001). Furthermore, they have documented hypogonadism (testosterone <300 ng/dl) rate is comparatively higher in US patients (26%, n=149) than the control group (7.5%, n=67) (p=0.002) [20].

Androgens have a beneficial role in preventing oxidative stress, chronic inflammation, and fibrosis [21]. Peri-urethral tissue analysis has proved the presence of androgen receptors. Androgen also maintains peri-urethral vascularity and trophicity of urethral mucosa. Hofer et al. have demonstrated the influence of androgen in maintaining periurethral vascularity [10]. They have found a significantly low expression of androgen receptors in the periurethral tissue of stricture patients with low blood testosterone [10]. Levy et al. examined pathological urethral tissue in LS and non-LS patients. After examining 81 pathological specimens of US, they concluded loss of androgen receptors in 43% (n=81) of patients with stricture [22]. Another prospective study by Gerbie et al. exhibited improved peri-urethral vascularity in testosterone and estrogen-supplemented castrated rats than non-supplemented castrated rats [23]. The evolution and outcome of urethral stricture are adversely affected in the background of low testosterone levels.

The main limitation of our study is the small sample size, with only six months of follow-up. Testosterone levels according to race, co-morbidities, smoking and genetic factors were not considered. The selection of cases was not stratified with possible etiologies like BXO changes, urethritis, and idiopathic. We only have demonstrated a statistical association between stricture severity, length, and recurrence with low testosterone levels. Causes and clinical symptoms of low testosterone were beyond the scope of this study.

## Conclusions

Our study distinctly demonstrates the evidence of low serum testosterone levels in urethral stricture patients. Low testosterone is related to more severe and long-segment stricture with an increased risk of recurrence. Further research is indeed essential to justify the significance of androgen levels in urethral stricture with a larger sample size. Future studies should enlighten more on the therapeutic significance of testosterone in urethral stricture. A combination of surgical therapy with testosterone supplementation urethral stricture may open a new window of treatment.
